# Can Traditional Chinese Medicine Diagnosis Be Parameterized and Standardized? A Narrative Review

**DOI:** 10.3390/healthcare9020177

**Published:** 2021-02-07

**Authors:** Luís Carlos Matos, Jorge Pereira Machado, Fernando Jorge Monteiro, Henry Johannes Greten

**Affiliations:** 1 Faculdade de Engenharia da Universidade do Porto, 4200-465 Porto, Portugal; fjmont@fe.up.pt; 2 CBSIn—Centro de Biociências em Saúde Integrativa, Atlântico Business School, 4405-604 Vila Nova de Gaia, Portugal; jmachado@icbas.up.pt; 3 CTEC—Centro Transdisciplinar de Estudos da Consciência da Universidade Fernando Pessoa, 4249-004 Porto, Portugal; 4 ICBAS—Institute of Biomedical Sciences Abel Salazar, University of Porto, 4050-313 Porto, Portugal; heidelbergschool@aol.com; 5 INEB—Instituto de Engenharia Biomédica, Universidade do Porto, 4200-135 Porto, Portugal; 6 German Society of Traditional Chinese Medicine, 69126 Heidelberg, Germany

**Keywords:** traditional Chinese medicine, TCM diagnosis, tongue diagnosis, pulse diagnosis, electrophysiology, acupoints, acupuncture

## Abstract

The integration of Traditional Chinese Medicine (TCM) in Western health systems and research requires a rational communicable theory, scientific proof of efficacy and safety, and quality control measures. The existence of clear definitions and the diagnosis standardization are critical factors to establish the patient’s vegetative functional status accurately and, therefore, systematically apply TCM therapeutics such as the stimulation of reflex skin areas known as acupoints. This science-based conceptualization entails using validated methods, or even developing new systems able to parameterize the diagnosis and assess TCM related effects by objective measurements. Traditionally, tongue and pulse diagnosis and the functional evaluation of action points by pressure sensitivity and physical examination may be regarded as essential diagnostic tools. Parameterizing these techniques is a future key point in the objectification of TCM diagnosis, such as by electronic digital image analysis, mechanical pulse diagnostic systems, or the systematic evaluation of acupoints’ electrophysiology. This review aims to demonstrate and critically analyze some achievements and limitations in the clinical application of device-assisted TCM diagnosis systems to evaluate functional physiological patterns. Despite some limitations, tongue, pulse, and electrophysiological diagnosis devices have been reported as a useful tool while establishing a person’s functional status.

## 1. Introduction

Traditional Chinese medicine is an ancient medical system that gives emphasis to human body integrity and regulation and its interrelationship with the surrounding environment [1,2]. The worldwide use of TCM practices has raised the question of how its integration in Western healthcare systems and research could be practically managed. This has encouraged the development of research strategies and the publication of a considerable number of scientific studies on the mechanisms and effects of TCM therapeutics [3,4,5,6,7,8,9,10,11,12,13,14,15,16,17]. Nevertheless, there is still much anecdotal evidence demanding appropriate study designs and sample dimensions to break down the resistance within mainstream conventional medicine [18]. The integration of TCM is a bidirectional process that requires the following preconditions [19]:

1. A rational communicable theory;
2. Scientific proof of efficacy and safety;
3. Measures of quality control.

Contemporary TCM could be understood as a model of vegetative system biology centered on the theory of regulation and the so-called guiding criteria as categories of clinical signs based on four physiological models of body regulation. These involve signs of neurovegetative origin felt as “fullness” or “emptiness”, signs of humoro-vegetative origin felt as “heat” or “cold”, signs of neuro-immunological stages expressed as “exterior” or “interior” affections, and signs of structural deficiency versus primary regulatory deficiency understood as “yin yang”. In this context, TCM diagnosis is a way of establishing the body’s vegetative functional status.

This integrated neurovegetative approach, complementary and interactive with Western medicine, translates to Western physiology the structural concepts of TCM language, making possible, therefore, the inherent rational use of reflex therapeutic systems, anti-inflammatory mechanisms, and mental training, which are applied in techniques such as acupuncture and “Qigong” [20,21]. This methodology not only requires a science-based conceptualization but also a standardization effort that goes from diagnosis to therapeutics [19]. The development of pragmatic and reliable technological systems to measure TCM related effects and even to calibrate and mimic skillful diagnosis procedures such as tongue and pulse diagnosis is critical.

One of the problems involved in operating research protocols and evaluating acupuncture success consists of rationally establishing the diagnosis by a systematic categorization of signs of symptoms. A proper diagnosis followed by a coherent intervention is the recipe to achieve positive results, as can be seen, for example, in the study conducted by Maimer et al. (2013) on the effects of needle acupuncture on the pain-induced reduction of lung function after median sternotomy, or even in the study conducted by Karner et al. (2013) on a repeated measures, double-blinded, and placebo-controlled, multicenter trial with 113 patients suffering from chronic osteoarthritis of the knee. This latter compared the effects of three acupuncture modalities (sham, semi-standardized modern, and individualized classical, based on Chinese medical diagnosis as defined by the Heidelberg Model of TCM) within two parameters: joint mobility and pain [22].

The previously mentioned studies show that acupoints’ diagnosis-dependent selection contributed to a higher analgesic effect. If acupoints are understood as reflex points that elicit specific neurovegetative changes, it can be speculated that they might only yield satisfactory treatment results if they match the current vegetative status of the patient [23]. In some cases, the variability between practitioners’ evaluations must be considered [24,25,26]. This variability, often related to the subjectivity of the variables and subcategories of diseases, can lead to an incorrect selection of acupoints and erroneous therapeutic results. Moreover, non-individualized, non-diagnosis based interventions could negatively contribute to some acupuncture meta-analysis, which based on the poor results of some inaccurate trials, evaluate acupuncture treatments as not being statistically different from controls [27]. Furthermore, acupoints’ exact location is critical and might significantly influence the results. In addition to lab-scale experiments involving nitric oxide biocapture over the skin in zones corresponding to acupuncture points [28], advanced technological methods such as magnetic resonance imaging (MRI), functional MRI, synchrotron radiation phase-contrast X-ray CT imaging, and positron emission tomography might help clarify the meridian network and validate the traditional “cun” measurement unit often used to locate acupoints [29,30].

The concept of syndrome or pattern in TCM (known as “Zheng”) is crucial while establishing the diagnosis [31]. It entails the human body’s overall physiological and pathological pattern as a function of a particular condition, which usually reflects an internal unbalance defined by a systematic analysis of the clinical symptoms and signs gathered by a practitioner [1]. Thus, to establish a general picture of the patient condition involving subtle and physical factors related to inner nature [32], pathological agent, and physiological status, Chinese medicine diagnosis considers four main essential examinations: looking, listening-smelling, palpating, and questioning [33]. During this process, tongue diagnosis is one of the aspects evaluated while looking, whereas pulse diagnosis is evaluated while palpating.

## 2. Tongue Diagnosis

In Chinese medicine, the tongue shows the state of some fundamental substances known as “qi” (commonly referred to as “vital force” or “energy”), “xue” (blood), and “jin ye” (body fluids) and correlates with groups of diagnostically relevant signs, which indicate the state of a body island, as well as with the functional properties of meridians [34]. TCM holds that “qi” and “xue” are functional powers in the body, or vegetative capacities to function, which may be seen in functional physical signs related with measurable physiological processes and aspects such as the increase of the peripheral microcirculation and skin temperature and the changes on acupoints’ electrical potential and resistance, and even on the surrounding biomagnetic field [9].

**Figure 1:**
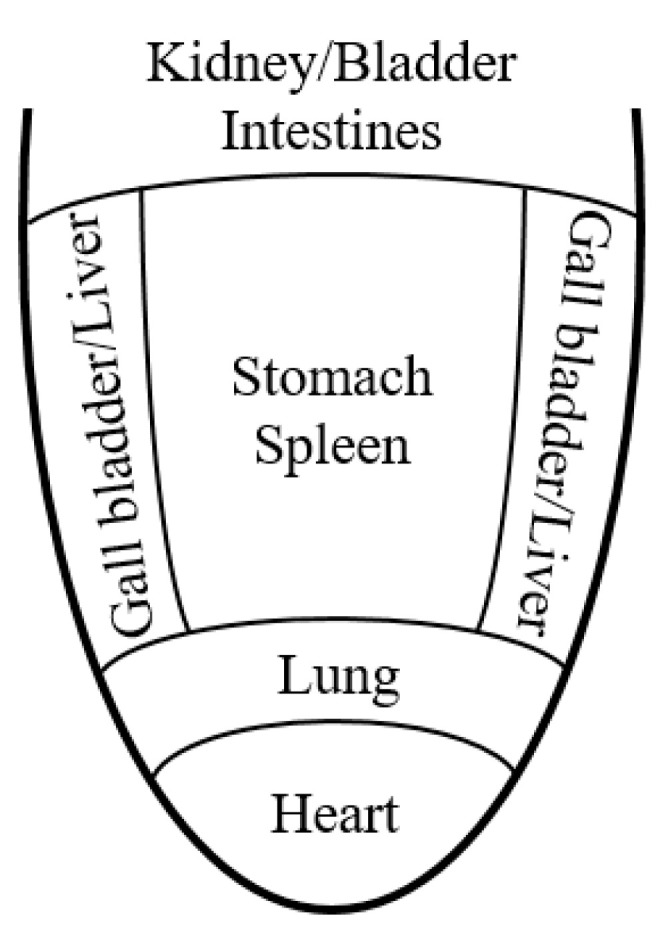
Tongue somatotopy in Chinese medicine diagnosis.

The tongue is a mirror for the internal organs, reflecting the body’s physiological and clinical-pathological condition (Figure 1). The color, size and form, motion, substance, coating, and geometric shape of the tongue, as well as changes in the tongue body, such as thickness, cracks, and teeth marks, are just a few qualities that must be evaluated and related to the health state of the patient [35].

**Figure 2:**
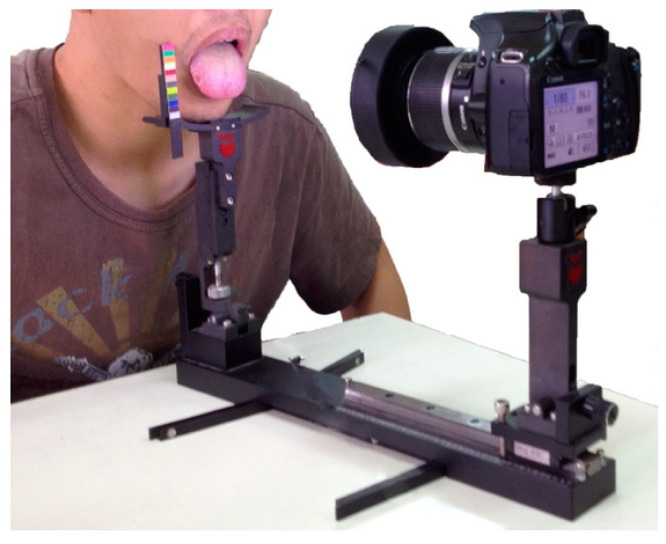
Illustration of tongue image capturing with the Automatic Tongue Diagnosis System (ATDS). Reprinted from ref. [39].

Tongue diagnosis requires proper skills and specific light conditions. Despite the difficulty, mostly because the diagnosis relies on the practitioner experience and knowledge, several approaches based on digital image capture and computerized tongue examination systems have been proposed and used in large scale studies, demonstrating its applicability for health identification and disease differentiation [35,36,37,38]. The Automatic Tongue Diagnosis System (ATDS, Figure 2) comprises hardware responsible for the image acquisition and software that allows the tongue body segmentation, feature extraction, and diagnosis. The hardware mainly integrates digital cameras equipped with charge-coupled devices (CCD) or complementary metal-oxide-semiconductor sensors, light sources such as light-emitting diodes (LED), cold-light halogen lamps, tungsten halogen lamps, and a color checker module embedded inside the digital image providing a color reference to a calibration model. After the tongue image is captured, the image-processing software, based on advanced statistical methods and mathematical models, allows the color correction, the tongue segmentation, and classification according to the extracted diagnosis parameters [35].

Tongue segmentation is a complicated procedure that deals with structural difficulties such as the variability of pathological details on the tongue surface and the diversity of tongue body shapes [40]. It requires robust and accurate algorithms, based on region or edge approaches to detect tongue boundaries.

The active contour model (ACM) or snake is an edge segmentation technique frequently used in tongue segmentation. However, it presents poor convergence of concavities, limited capture range, undesirable contracting and clustering effects, and lack of global control [35,41]. Several methods have been proposed in combination with the ACM to overcome these limitations, such as the bi-elliptical deformable template, the watershed transform, the gradient vector flow, and the knowledge-based initial tongue body boundary detection plus color gradient [40,41,42,43]. Other techniques are also able to be successfully used in tongue segmentation, such as the threshold method using the Otsu’s thresholding algorithm, the color control-geometric and gradient flow snake, and the double geo-vector flow [44].

The color and coating of the tongue body are the two main criteria used in the development of ATDS. Features such as tongue shape, fissures, ecchymosis, tooth marks, and red dots can also be considered by some systems [45]. A bluish tongue, petechiae, and engorged sublingual collateral vessels are potential tongue manifestations of blood stasis, which, according to Hsu et al. (2016), is a relevant predictor of diabetes mellitus [46]. Indeed, some practitioners integrate sublingual veins inspection during their tongue diagnosis routine. Chiu et al. (2002), as well as Yan et al. (2009), found a good correlation between the results of computerized image analysis of the patient’s sublingual veins and their physiological and pathological conditions [47,48].

As a general requirement, the developed ATDS must be able to mimic, in a reproducible way, the traditional tongue diagnosis, and for that, when submitted to clinical tests, their output must correlate with the findings of experienced TCM practitioners. In a study on the agreement between an automatic tongue diagnosis system and the evaluation made by several TCM practitioners, ATDS was shown to be more consistent with significantly higher intra-agreement than TCM doctors. Moreover, the inter-agreements between the ATDS and TCM doctors were both moderate [39]. Similar results were found by Kim et al. (2012) while evaluating the tongue coating thickness [49]. Those positive results agree with clinical studies designed to access the tongue color and coating in patients suffering from functional dyspepsia, gastritis, diabetes, colorectal, and lung and breast cancers [46,50,51,52,53,54,55].

Despite the encouraging results, each methodology’s specificities may lead to inconsistencies in the practical clinical application [35]. Nevertheless, researchers have been interested in the ATDS as a tool to standardize traditional tongue diagnosis and for educational purposes [56,57]. Some have seen the advances in smartphone technology as an opportunity to create immediate diagnosis applications; however, there is still much work to do to overcome obstacles and limitations such as the environmental lighting conditions and the existence of standard artificial illumination with temperature control, the low image resolution and absence of color correction of some systems, and the correlation with well-established diagnosis [35,58].

## 3. Pulse Diagnosis

**Figure 3:**
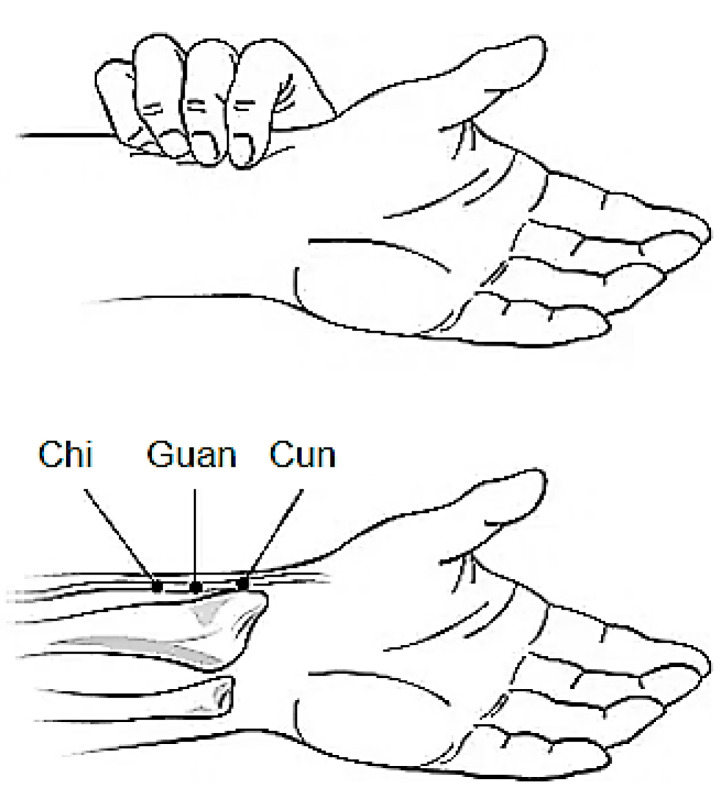
Pulse diagnosis gauge sites over the radial artery. Reprinted with permission from ref. [60], Copyright 2017 Cao Xuan cu.

The pulse (“mo”) in Chinese Medicine is considered the most important diagnostic technique, requiring a good knowledge of Chinese physiology to interpret the findings. Those can give detailed information on the state of the patient’s internal organs, as they reflect the patient’s flow of “qi” and “xue” and the “yin yang” character at the moment. The pulse is felt at the radial artery, which is divided into three areas: the pollical site at the front near the wrist, the clusal site at the middle, and the pedal site at the lower level (also known as “cun” or inch, “guan” or barrier, and “chi” or foot, respectively) (Figure 3) [59].

The pulse is taken on both sides at three palpable depths related with “qi”, “xue”, and organs [61]. The deeper level corresponds to the “yin” and at the superficial level to the “yang” physiological regulation aspects or, in another language, the deeper level to the “yin” organs (solid organs) and at the superficial level to the “yang” organs (hollow organs) [62]. The two-depth method is commonly taught in Australia, Europe, and the USA and is frequently used by practitioners of these locations [61]. The three areas of the wrist are related with the so-called “Triple Burners”, and so the front position corresponds to “Heaven” and reflects the diseases from the head to the chest; the middle position corresponds to “Man” and reflects the diseases from the diaphragm to the umbilicus; and the lower position corresponds to “Earth” and reflects the diseases from the umbilicus to the feet [63].

Pulse diagnosis requires sensitivity and special skills. To feel its subtle variations, each practitioner must learn how to identify them by experience and continuous practice. Despite its importance, palpation remains subjective and mysterious, standing as an obstacle in clinical practice and research [64]. Indeed, ancient TCM canons document dozens of pulse qualities including floating, sunken, slow, rapid, surging, fine, vacuous, replete, long, short, slippery, rough, string-like, tight, soggy, moderate, faint, weak, dissipated, hollow, drum skin, firm, hidden, stirred, intermittent, bound, skipping, and racing, to mention a few [65]. The amount of detail requires a unique perception, and even with practice, the variability in the diagnosis is considerable, making the intra- and inter-rater pulse diagnosis reliability not high between TCM practitioners [66].

The quantification of pulse patterns is an essential requirement for objectifying TCM pulse diagnosis. Arterial pulse waveform has been studied in order to develop quantification strategies [67,68,69,70,71,72]. In this process, researchers have used a wide range of sensors including piezoelectric sensors, piezoresistive strain gauges, magnetic sensors, liquid sensors, acoustic sensors and Doppler ultrasonic devices, infrared sensors, and optical and photoelectric sensors, among others [73]. Once the signal is acquired, processing and analysis strategies are necessary to interpret the Chinese pulse waveform patterns objectively. Shu et al. (2007) proposed the following four classification indices: the wavelength, the relative phase difference, the rate parameter, and the peak ratio. The relative differences between the mentioned indices allowed the authors to distinguish 13 pulses [74], while others have reported the differentiation of pulse qualities by using neural network strategies to classify patterns shown in pulse images [75].

Pulse taking can be performed by pressing with one finger in each gauge site or simultaneous palpation, which gives a trend for the whole body state. These two methods are used together to obtain full information; however, regarding different clinical viewpoints, some practitioners insist on using simultaneous pulse taking, while others use site-to-site evaluation. This stands as an obstacle in automatic pulse taking, and for that reason, to clarify this issue and find differences in these two approaches, Chung et al. (2013) conducted a study using a tactile array sensor. They found that both methods are positively correlated concerning the entire trend and specific pulse-taking depth, with no discrimination while checking the diagnosis patterns. Nonetheless, once site-to-site evaluation cannot simultaneously compare the three gauging sites, simultaneous pulse-taking seems to be the best option for a possible standard pulse-taking procedure [76].

**Figure 4:**
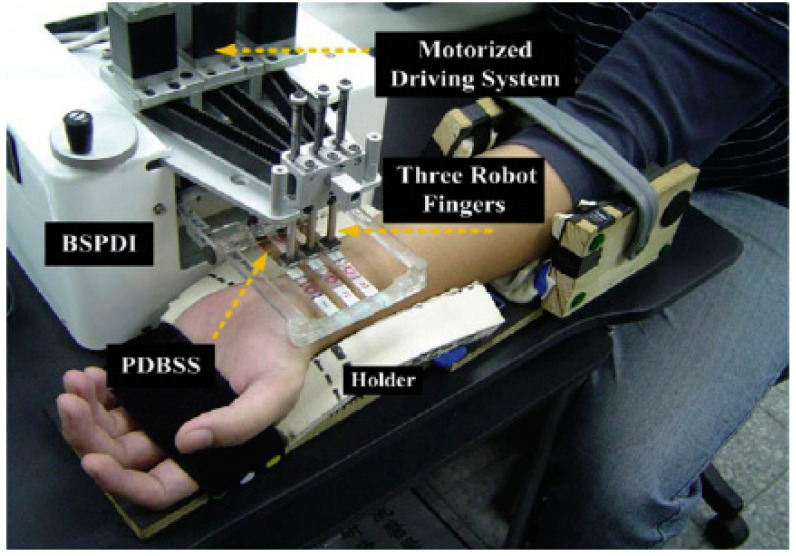
Bi-Sensing Pulse Diagnosis Instrument (BSPDI) equipped with a Pressure-Displacement Bi-Sensing System (PDBSS). Reprinted with permission from ref. [77]. Copyright 2012 Elsevier and Copyright Clearance Center.

Tactile array and pressure sensors have been used with success to detect some pulse particularities [64,76,77]. In this field, Luo et al. (2012) developed and tested a Bi-Sensing Pulse Diagnosis Instrument with a Pressure-Displacement Bi-Sensing System coupled to a robot finger system (Figure 4). This device was equipped with pressure sensors whose signals simulated the practitioner fingertip sensations. The authors found that the pulse signals obtained from the robot fingertips accurately represented the TCM practitioner’s fingertip sensations at the level of their finger-reading ability. These authors concluded that this method could be used to demonstrate the experienced finger-reading skills of a TCM practitioner and quantitatively record the inherent pulse findings [77].

Using the same type of technology, Chu et al. (2014) conducted a study to evaluate the pulse qualities at three positions and three depth levels. These authors found a relation between the measurements and the patterns expressed by a three-dimensional pulse mapping, representing the mimicking of the practitioner’s fingertip sensations [64]. Despite the effort, there is still a lack of studies exploring the pulse depth qualities and their relation with different pathological features. Chung et al. (2015) proposed a method that defines the pulse-taking depth based on the artery’s width, considering the initial touching position as the starting point and the artery obstructed position as the ending point. With this approach, the different depths are defined by the different percentages of the artery width [78].

The previous studies corroborate the validity of these devices in the parameterization of pulse diagnosis; however, more research is needed to improve the detection and quantification of subtle wall-dependent qualities and to overcome waveform artefacts related to the subject’s movement or breathing.

## 4. Electrophysiological Diagnosis Devices

For many years, researchers have claimed that acupuncture points are unique locations on the body surface at which the skin’s electrical conductivity is maximal compared with neighboring spots [79,80]. Moreover, the electrical properties of these “bioactive” points are also characterized by a reduced impedance and resistance, an increased capacitance and higher electrical potential compared with non-acupuncture points [81]. Once the conductivity between two points along the same conduit (or meridian) is greater than that between points not sharing this relationship, conduits could be considered pathways of lower electrical resistance throughout the body where electrical potentials are spread without needing to overcome the resistances of cellular membranes. These low-resistance extracellular pathways might be connected to the internal organs, thus providing a theoretical basis for the traditional Organ–Conduit associations [62].

**Figure 5:**
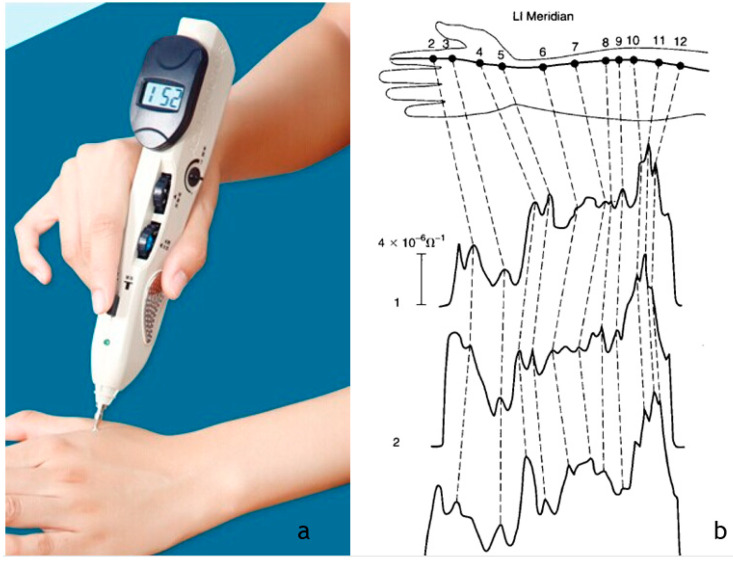
Acupoint detector device (**a**) [83] and three successive conductance scans along the Large Intestine Meridian–numbers in the upper limb correspond to acupoints (**b**) Reprinted with permission from ref. [62] Copyright 2002 Elsevier and Copyright Clearance Center.

Nowadays, several devices can measure the electrical resistance (related to the electrical conductance) in specific skin areas coincident with the acupuncture points [82]. An example of one of these devices can be seen in Figure 5a, as well as the electrical conductance, during three consecutive measurements along the large intestine conduit (LI meridian) (Figure 5b).

Some authors have shown that it is possible to distinguish between the conduit and non-conduit tissue by applying low-frequency electrical stimulation in the skin [84,85,86,87]. Based on this principle, several electrodermal screening techniques were developed not only for point location but also for diagnostic and therapeutic purposes [84,88,89,90,91,92]. The development of this kind of technology must consider critical technical issues that contribute to the final electrodermal reading and may cause doubts about the validity of these devices, such as the electrode polarizability, the stratum corneum impedance, the presence of sweat glands, the choice of contact medium, and the electrode geometry, among others [81].

An acupoint’s electrical potential is a relative quantity that quantifies how much energy capacity it possesses compared to a reference. It is a standard measure used to study bioelectricity related to low-level endogenic currents and assess functional effects and particularities along conduits. Lee et al. (2005) show that while studying the effects of acupuncture on the potential along the stomach conduit of healthy and unhealthy patients suffering from gastric disease, a diagnosis can be made by comparing the levels of potential difference and its regularity in the conduit [88]. Research has shown that the electrical potential differences can be used to monitor changes in the conduits and acupoints [93,94]. Acupoints’ states modify as a function of different stimuli, which can induce physiological changes and alter the endogenous electrical potential and current in the tissue [94,95]. As shown by Matos et al. (2019; 2021), “Qigong” practice, acupuncture and moxibustion can be used as stimuli to generate electrical potential changes in acupoints. Those changes seem to be according to TCM theory and may explain the vegetative physiological changes associated with “qi flow” in Chinese Medicine [96,97].

The Electroacupuncture According to Voll (EAV) and the Ryodoraku mechanism for measuring conduits’ electrical activity are two well-known systems. In the first one, the acupoint direct current (DC) resistance reflects the condition of the associated organs or systems and could diagnose and monitor the patient’s health. The diagnosis depends on measuring the relative electrical conductance (in a scale ranging from 0 to 100) and its time dependence. According to Voll’s calibration, readings from 50 to 65 (or resistance between 53 and 95 kΩ) are regular; readings above 65 (or resistance less than 53 kΩ) indicate inflammation, and readings below 50 (or higher than 95 kΩ) indicate degeneration in the organs associated with the measured point [98]. These devices measure the current intensity in a series circuit containing a voltage source (usually a few volts) and two resistors: one is the resistance between the electrode-acupuncture point and a cylindrical contact electrode kept firmly in one hand, and the other is usually chosen to represent the mean value of repeated measurements on acupuncture points of healthy persons [79]. EAV theory is the base for the standard electrodermal screening (EDS) techniques used to evaluate the electrical resistance. This technology is useful in diagnosing certain conditions such as upper gastrointestinal bleeding, kidney failure, or even cancer [99,100].

The Ryodoraku mechanism for measuring the electrical activity of conduits was developed by Dr. Yoshio Nakatani in the 1950s. Ryodoraku means good conductive line [101], and its output reflects the condition of specific organs by analyzing and comparing their mutual relations, considering the measurable changes in the electrical properties of some acupoints [102]. This technique chooses the Source (Yuan) acupoints located in the wrists and ankles, which are the places where the “yuan qi” (original “qi”) of the corresponding organs passes through abundantly, to represent the respective conduits. Some studies show that this system could investigate changes in physiological and psychological variables of subjects submitted to different stimuli [103,104]. Moreover, Ryodoraku is a supplementary diagnostic method of renal colic [102], and a useful tool for evaluating the therapeutic effects of acupuncture and related techniques in treating certain conditions such as back pain, gall-bladder conduit dysfunction, and obesity [101,105,106,107].

Although much of these findings corroborate the assumption that conduits act as a preferred pathway for electrical stimulus conduction, more studies are needed to ensure reproducibility and reliability, overcoming potential misleading factors such as the electrode material, size and shape, pressure exerted by the probe, duration of probe application, the inclination of the probe tip on the skin, and variations in skin condition [108].

## 5. Conclusions

The integration of TCM in Western health systems and research requires a profound evaluation of effectiveness and security, which has inherent to it a science-based conceptualization and standardization effort, that goes from diagnosis to therapeutics. The systematic assessment of TCM diagnosis variables is essential in developing new technological systems that can calibrate and mimic complex and skillful diagnosis procedures such as tongue and pulse diagnosis.

Besides pulse and tongue diagnosis, TCM diagnosis integrates other components that allow the practitioner to establish a general picture of the patient condition, involving subtle and physical factors related to its inner nature, pathological agent, and physiological status, known as the constitution, the agent, and the orb, respectively. For example, the constitution represents a person’s tendency to predominantly express the clinical manifestation of a Phase or relevant functional signs (orb pattern). In other words, the constitution reflects the individual functional properties and the person’s inner nature based on the phenotype. As described in the Yee Wong review (2020), the constitution integrates aspects such as physical structure, physiological function, pathological reaction, psychological or mental stability, and metabolism [109]. The evaluation involves, for example, observing the body complexion, motion and language, and the tone of the voice and cadence of the speech. The parameterization of these variables using technological systems seems to be a complex but not impossible process. Image and sound systems with advanced data analysis and algorithms might contribute to the standardization process.

This review, based on PubMed, ScienceDirect, and Google Scholar searches, mainly included research papers and reviews focused on using technological methods to quantify variables related to tongue and pulse diagnosis and electrophysiological properties of acupoints. As shown, a development effort has been made over the last decades, with a broad research outcome covering TCM fundamental concepts and new diagnosis devices’ clinical applications. Although some difficulties and limitations are pointed out in this review, the positive results have encouraged commercial investment and the onset of new systems used by many practitioners as a complementary diagnostic tool.

## Data Availability

Not applicable.
